# Surufatinib related nephrotic syndrome in a pancreatic neuroendocrine tumor: a case report and review of literature

**DOI:** 10.3389/fonc.2025.1546217

**Published:** 2025-05-21

**Authors:** Chengqian Shi, Jinglian Lin, Xiadan Xiang, Wenlong Bao, Jie Yang

**Affiliations:** ^1^ Department of Nephrology, The Second Affiliated Hospital of Zhejiang Chinese Medical University, Hangzhou, China; ^2^ Department of Traditional Chinese Medicine, The Cancer Hospital of University of Chinese Academy of Sciences (Zhejiang Cancer Hospital), Hangzhou, China

**Keywords:** pancreatic neuroendocrine tumors, IgA nephropathy, nephrotic syndrome, surufatinib, podocyte

## Abstract

Pancreatic neuroendocrine tumors (pNETs) are rare, heterogenous neoplasms originating from pancreatic neuroendocrine cells, which regulate hormone secretion and metabolic homeostasis. Surgery is the primary method of control and potential cure for pNETs and targeted therapies have also been investigated for low-grade inoperable or distant metastatic pNETs. Surufatinib, an oral angio-immuno kinase inhibitor, is approved for treating inoperable or late-stage, low-grade (G1 and G2), well-differentiated pNETs and extrapancreatic neuroendocrine tumors. This study describes a case of nephrotic syndrome in a middle-aged woman with pNETs. This patient showed nephrotic syndrome after surufatinib treatment 3 months and renal pathology suggested IgA nephropathy with moderate podocyte injury. However, the nephrotic syndrome was relieved after 2 weeks of discontinuation of surufatinib. After resuming treatment with low-dose surufatinib for 2 weeks, the random proteinuria quantification was increased and the proteinuria turned negative after discontinuation of surufatinib again. It provides a reference for surufatinib related nephrotic syndrome in patients with pNETs and the potential mechanism between surufatinib and podocyte injury in IgA nephropathy needs to be investigated.

## Introduction

Pancreatic neuroendocrine tumors (pNETs) are relatively rare malignancies with highly heterogeneous biological behavior and diverse clinical manifestations (1). The annual incidence of pNETs from 2000–2012 was 0.48 per 100000 persons and the median overall survival (OS) was 3.6 years (2). Surgery is the primary treatment for improving the prognosis of pNETs, but the combination of systemic drug therapy combined with local treatment should be adopted, for patients with distant metastases or locally advanced that cannot be surgically removed at low to intermediate grade (Grade 1 or Grade 2, G1 or G2).

Surufatinib is an oral angio-immuno kinase inhibitor that selectively targets vascular endothelial growth factor (VEGF) receptors 1-3, fibroblast growth factor receptor type 1 (FGFR1), and colony stimulating factor-1 receptor (CSF-1R) (3). Surufatinib showed promising antitumor activity and improved progression-free survival with an acceptable safety profile in Chinese patients with progressive, advanced pNETs in the phase III SANET-p trial (NCT02589821) (4). Currently, surufatinib has been approved for treating pNETs and extrapancreatic neuroendocrine tumors (epNETs) in China (5).

In this study, we report a case of a 47-year-old woman suffering from pNETs, with the treatment of surufatinib for 3 months. The patient clinically showed nephrotic syndrome and Immunoglobulin A (IgA) nephropathy with moderate podocyte injury in renal pathology.

## Case presentation

**Figure 1:**
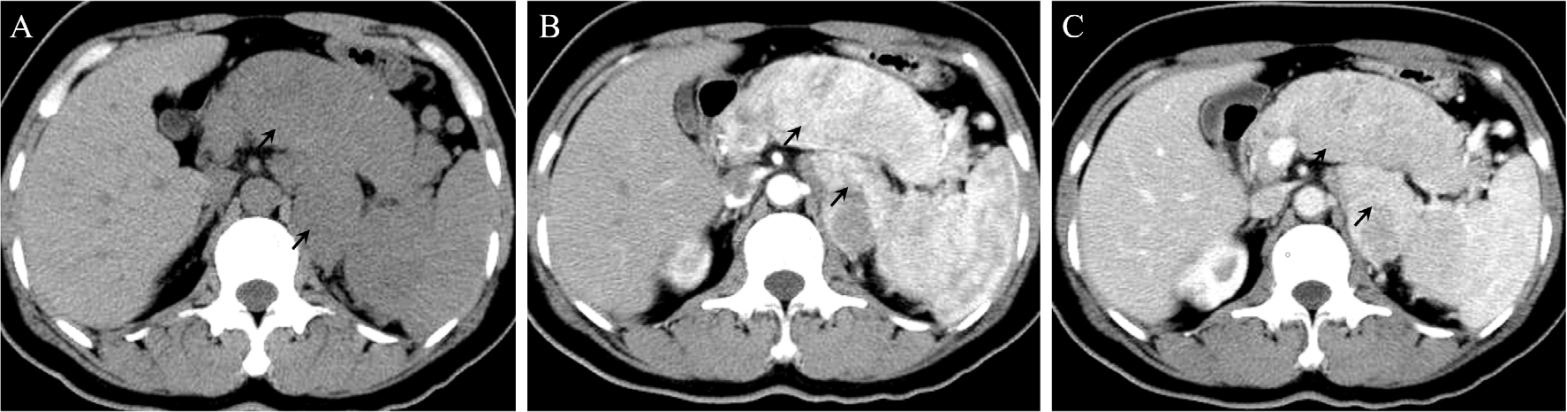
**(A)** On a plain CT scan, the shape of the body and tail of the pancreas was thickened, with a huge mass of about 12 * 4cm. **(B)** In the arterial phase, the lesions showed significant enhancement. Multiple enlarged lymph nodes can be seen in the left adrenal region and the inner courtyard of the spleen. **(C)** In the venous phase, the enhancement of the lesions decreased.

**Figure 2:**
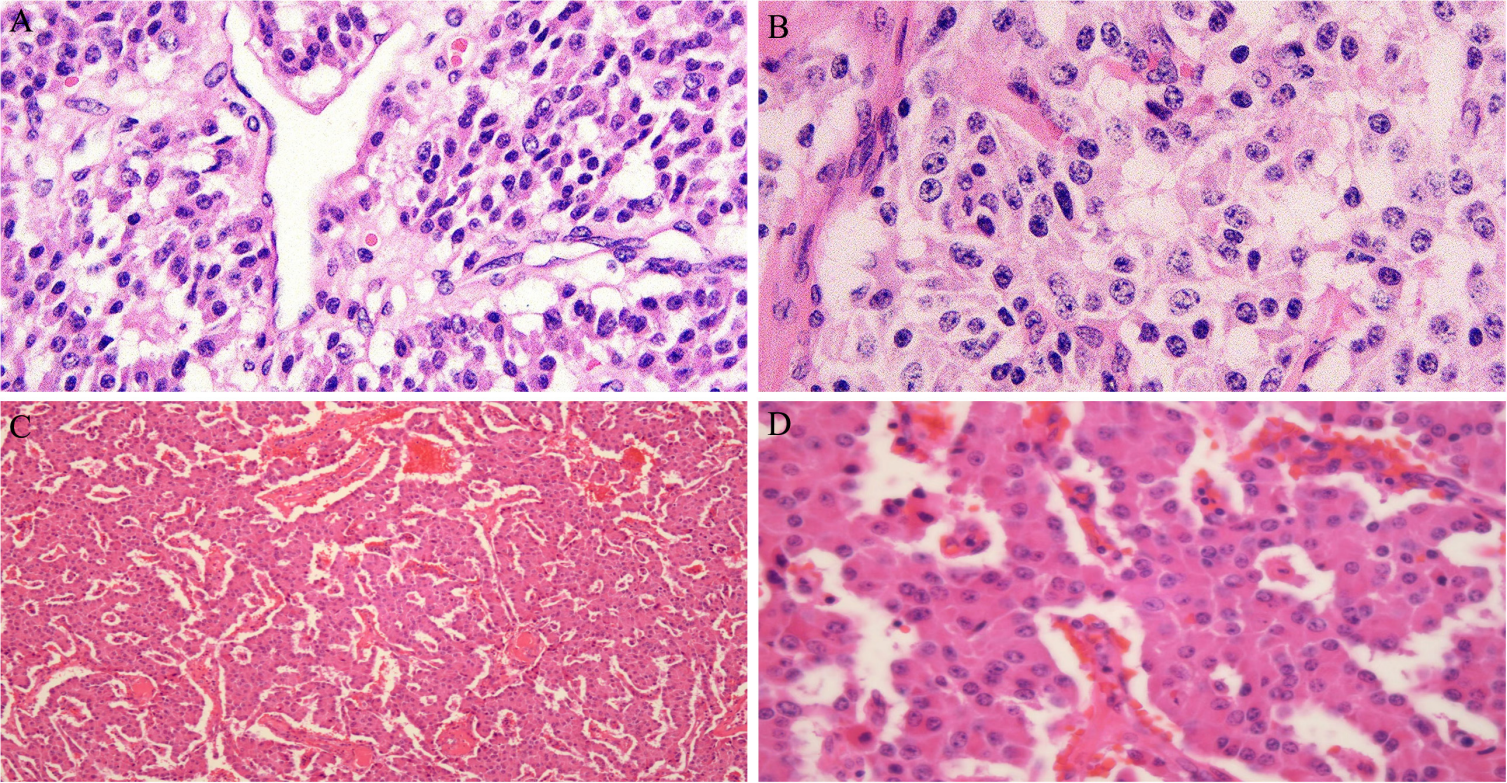
**(A, B)** Pancreatic neuroendocrine tumors, immunohistochemistry and oncogene detection: CAM5.2(+), PR(++,80%), AAT(+), Vim(-), CD10(-), Catenin\β-Catenin(+)、CD117\c-kit(-), Ki-67(+, 2%). **(C, D)** Neuroendocrine tumor found in liver tissue and considering pancreatic origin based on medical history, immunohistochemistry: Hepa(-), AFP(-), CK19(-), CK20(+), CgA(+), Sy(+), CD56(+), Ki-67(+, 5%), P53(+), SSTR2(+), CD10(-), β-Catenin(+).

In 2016, a 47-year-old woman found pancreatic abnormalities during a physical examination without any clinical manifestations. Abdominal computed tomography (CT) showed that the shape of the body and tail of the pancreas was thickened, with a huge mass of about 12 * 4cm and uneven density with CT value of 41Hu (**Figure 1A**). Abdominal enhanced CT showed continuous enhancement of pancreatic mass, with multiple enlarged lymph nodes visible in the left adrenal and spleen areas (**Figures 1B, C**). The rest of the patient’s examination and laboratory indicators were normal. Combined with her imaging characteristics, laboratory indicators and clinical manifestations, it was considered that the huge mass might be a pancreatic malignant tumor. In May 2016, the patient underwent surgical resection of a huge pancreatic mass, splenectomy, and unilateral adrenalectomy. Postoperative pathology (Pathological ID: 201612356) suggested a pancreatic neuroendocrine tumor (G2, 8*7*6 cm) (**Figures 2A, B**). Based on the above medical data, the patient was diagnosed with a non-functional pancreatic neuroendocrine tumor (pNET).

**Figure 3:**
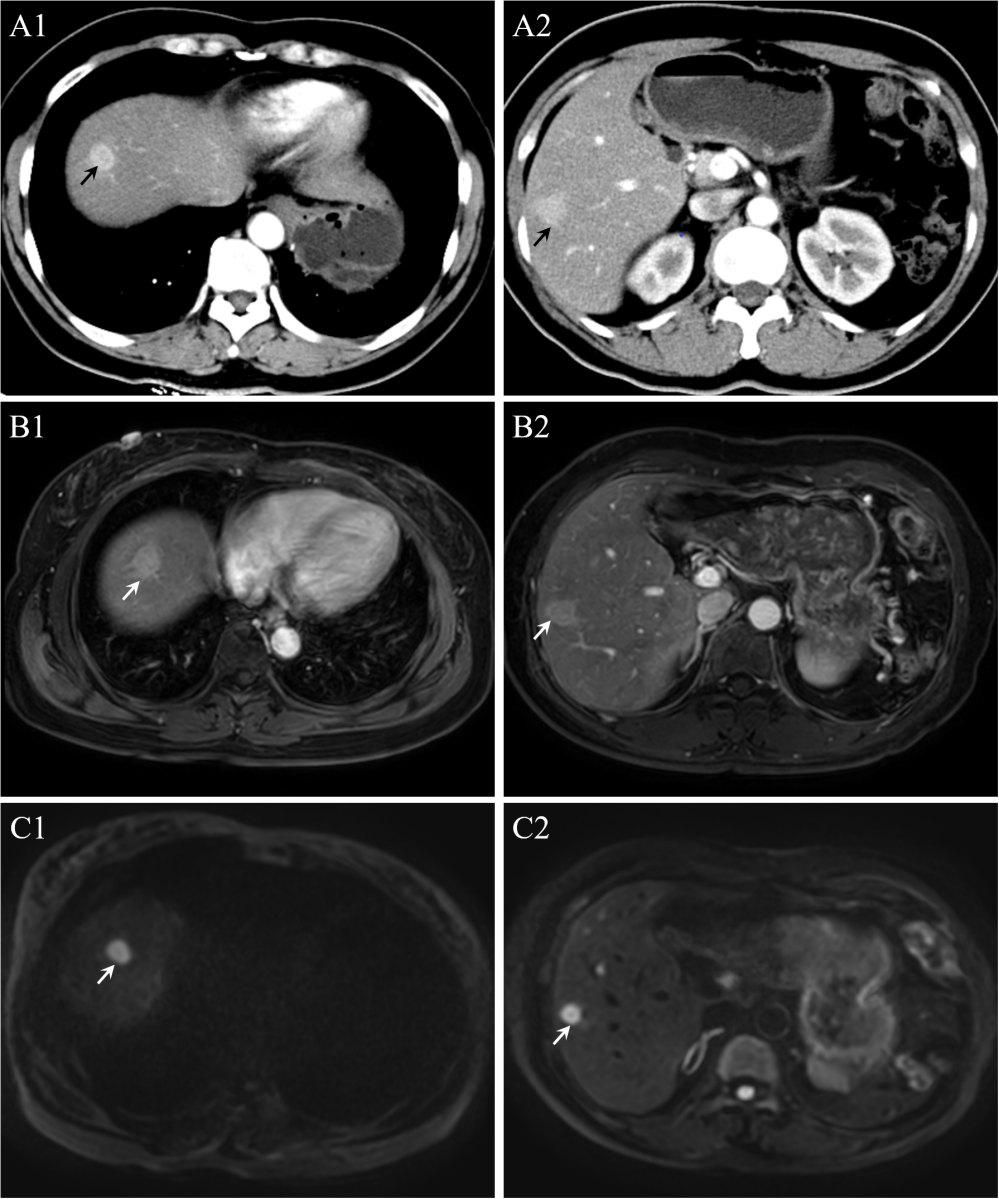
**(A)** In the arterial phase, multiple nodules in the liver. **(B, C)** On a magnetic resonance imaging and diffusion weighted imaging, multiple abnormal signals are detected in the liver.

In February 2023, ​the patient returned to the hospital for a re-examination and an abdominal CT revealed multiple nodules in the liver. Liver enhanced magnetic resonance imaging indicated multiple abnormal signals in the liver and, in combination with medical history, metastatic tumors are considered (**Figures 3A-C**). Subsequently, the 5th, 6th, 7th, and 8th segments of the liver were surgically removed. Postoperative pathology (Pathological ID: 202307422) showed neuroendocrine tumors were found in liver tissue and the pancreas origin was first considered (**Figures 2C, D**). Then, in April of the same year, ultrasound-guided liver microwave ablation was performed.

Given the risk of multiple metastases and malignancies, the surufatinib 300 mg orally once daily was initiated in the third week after cytoreductive surgery. Previously, the patient had hypertension for more than seven years and diabetes for more than four. At present, oral nifedipine controlled-release tablets of 30 mg once daily and metformin hydrochloride tablets of 200 mg once daily were used, the blood pressure and blood glucose were generally under control.

**Figure 4:**
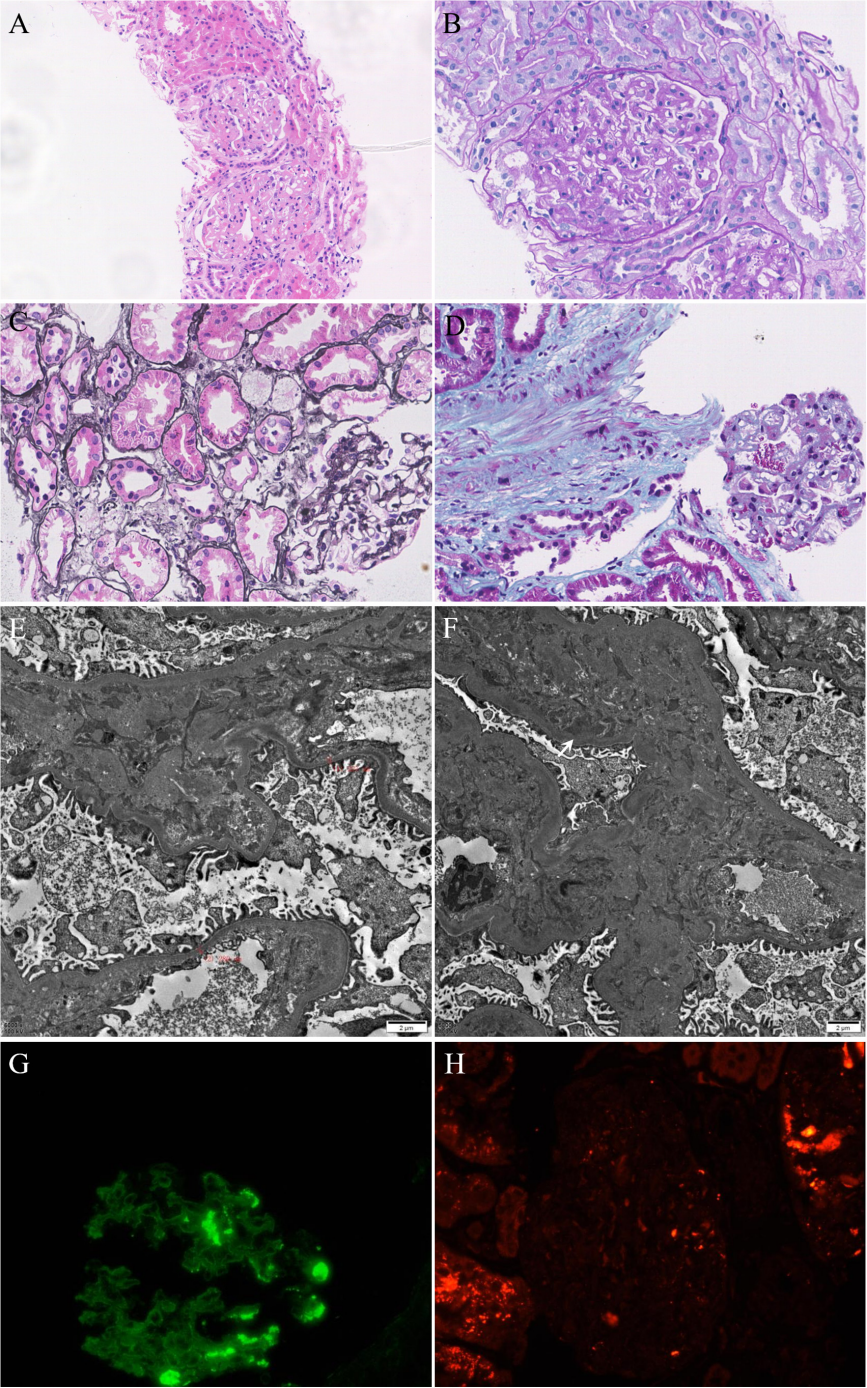
**(A–C)** Mild mesangial hyperplasia, mild interstitial fibrosis and tubular atrophy with a small amount of inflammatory cell infiltration. **(D)** Masson staining showed the deposition of fuchsinophilic proteins in mesangial area. **(E)** Mild widening of the mesangial area and partial fusion of the foot processes (60%). **(F)** Deposition of electron dense in the mesangial area. **(G)** IgA(+++), segment deposition. **(H)** KM55(-).

After 3 months of treatment, the patient gradually developed edema in both lower limbs, sometimes accompanied by facial edema, without chest tightness, shortness of breath, nausea, vomiting, or other symptoms. We conducted a preliminary examination and found that proteinuria was positive (+ +) in the urine routine. Further evaluation found severe proteinuria (Urinary microalbumin, UMa 1873.2 mg/L, 24-hour total protein 3184.5mg/24h and urinary microalbumin/creatinine ratios, ACR 264.3mg/gCr), hypoproteinemia (albumin, Alb 26.4g/L) and hyperlipidemia (triglyceride 2.25mmol/L). We diagnosed the patient with nephrotic syndrome based on clinical manifestations and laboratory indicators. To clarify the patient’s renal lesions and possible causes, we arranged an ultrasound-guided renal biopsy. Renal pathology suggests IgA nephropathy (KM55 negative) with moderate podocyte injury (**Figures 4A-H**). Considering the atypical deposition of IgA antibodies in renal pathology, the more significant podocyte lesions, and the patient’s medical history, we consider this to be tumor or drug related nephropathy. Therefore, we discontinued surufatinib and adjusted the antihypertensive drugs (sacubitril valsartan sodium tablets 100mg twice daily) and hypoglycemic drugs (empagliflozin tablets 10 mg once daily) after communicating with the patient. After two weeks of treatment, the patient’s edema symptoms and proteinuria urinalysis were significantly relieved (UMa 168.1 mg/L, ACR 103.1 mg/gCr) accompanied by an increase in serum albumin (Alb 32.7g/L). Based on the consideration of pNET, the oncology department recommended that the dose be reduced and the patient re-started surufatinib (200 mg once daily) treatment, every 4 weeks as a course. Regular monitoring of patient urinalysis showed a significant increase in random proteinuria quantification (UMa 738.7 mg/L) after half a course of treatment. Finally, the patient discontinued the surufatinib treatment on our recommendation, and the urine protein was negative after multiple examinations (UMa 86.8 mg/L, ACR 21.8 mg/gCr, **Supplementary Figure 1**). Antitumor therapy was temporarily suspended based on multidisciplinary team consensus and patient preference, with rigorous monitoring of tumor status and hepatic/renal function parameters. A predefined escalation protocol was established to initiate alternative targeted agents (e.g., everolimus) or chemotherapeutic regimens should disease progression occur.

## Discussion

We report a case of a pNET patient treated with surufatinib. Nephrotic syndrome onset occurred 3 months after the surufatinib treatment and ameliorated after surufatinib cessation. Unexpectedly, renal biopsy revealed IgA nephropathy without TMA.

pNETs can be divided into functional or nonfunctional pNETs, based on the ability to elaborate active hormones, which cause various clinical symptoms, such as hypoglycemia, watery diarrhea, and severe ulcer diathesis (1, 6). The incidence of nonfunctioning pNETs is increasing, but patients with resected functional pNETs have a better OS compared to patients with nonfunctional pNETs, and a considerable proportion of functional pNETs may be detected at earlier stages because of obvious clinical symptoms (1). However, nonfunctional pNETs can also be associated with nonspecific clinical symptoms, such as obstruction of the digestive tract, jaundice, and pancreatitis, caused by tumor compression of the pancreaticobiliary duct or invasion of peripancreatic organs. pNETs are characterized by multiple clinical behaviors, which can range from small benign lesions or slow-growing indolent tumors to invasive lesions or widespread metastases (6).

Actually, many solid and hematologic malignancies are associated with kidney disease, such as membranous nephropathy (MN), Minimal change disease (MCD), IgA nephropathy, focal segmental glomerulosclerosis (FSGS), and other pathological types of glomerular diseases (7, 8). MN is the most common pathological type in patients with solid tumors, particularly in lung and gastric cancers, sometimes in renal cell carcinoma (RCC), prostate cancer, and thymoma (9). In comparison, MCD has been reported in association with lung cancer, classic Hodgkin lymphoma, colorectal cancer, RCC, and thymoma and IgA nephropathy can be associated with RCC, and tumors of the respiratory tract, nasopharynx, and buccal mucosa (8, 10). However, the exact pathogenesis in most cases remains uncertain. Several studies have reported the association between pancreatic cancer and kidney diseases such as MN, MCD, and IgA nephropathy (7, 8), but few studies have reported the association between pNETs and glomerular diseases or the related pathological mechanisms, it may manifest with RCC and pNETs in patients with von Hippel - Lindau (VHL) disease (11, 12).

Significantly, not only the tumors itself can cause kidney disease, but the newly developed or conventional anticancer chemotherapeutic agents are nephrotoxic and can damage the kidney directly or indirectly, which can affect morbidity and mortality in patients with cancer (7). Anticancer chemotherapeutic agents can injure all nephron segments and the degree of damage is always related to dose and duration. For example, cisplatin is an effective conventional anticancer chemotherapeutic agent that is widely used against a variety of solid tumors, but its clinical use is often limited due to nephrotoxicity, especially in the proximal tubular epithelial cells (PTECs), which the pathological mechanisms include DNA damage, mitochondrial pathology, oxidative stress and other stress responses (13).

Surufatinib was first approved in China for the treatment of late-stage, well-differentiated, epNETs on 30 December 2020 and pNETs later (3). Previous trials and systematic reviews had reported the efficacy of surufatinib on epNETs and pNETs, significantly improving progression-free survival in patients (4, 5, 14, 15). However, the safety of newly developed anticancer agents is also the key point affecting clinical use. Most participants had not less than one treatment-related adverse event during the study [96% [108/113] in the surufatinib group vs. 92% [54/59] in the placebo group in SANET-p study (4), and 98% [126/129] in the surufatinib group vs. 96% [65/68] in the placebo group in SANET-ep study (5)], primarily of grade 1 or 2. Surufatinib-related serious adverse events were reported in 22% [22/113] in the SANET-p study (4) and 25% [32/129] in the SANET-ep study (5) compared with in the placebo groups, 7% [4/59] and 13% [9/68], respectively. The most common surufatinib-related adverse events of grade 3 or worse were hypertension [38% [43/113] in the surufatinib group vs. 7% [4/59] in the placebo group in SANET-p study (4), and 36% [47/129] in the surufatinib group vs. 13% [9/68] in the placebo group in SANET-ep study (5)], and proteinuria [10% [11/113] in the surufatinib group vs. 2% [1/59] in the placebo group in SANET-p study (4), and 19% [25/129] in the surufatinib group vs. 0% [0/68] in the placebo group in SANET-ep study (5)], respectively. Meanwhile, combining all grades of adverse events, the major reason caused dose interruption or reduction was proteinuria [22% [25/113] in the surufatinib group vs. 2% [1/59] in the placebo group in SANET-p study (4), and 29% [38/129] in the surufatinib group vs. 1% [1/68] in the placebo group in SANET-ep study (5)] and hypertension (14% [16/113] in the surufatinib group vs. 2% [1/59] in the placebo group in SANET-p study (4), and 16% [20/129] in the surufatinib group vs. 1% [1/68] in the placebo group in SANET-ep study (5)). To a certain extent, proteinuria has become one of the important adverse events affecting the clinical application of drugs and the prognosis of patients with NETs.

It is well known that proteinuria is one of the key biomarkers of kidney injury, indicating disruption or dysfunction of the glomerular filtration barrier (GFB). The breakdown of GFB may present with nephrotic syndrome (proteinuria, edema, hypoalbuminemia, and hyperlipidemia), proteinuria with hypertension, or isolated proteinuria in clinical. In the mechanism, GFB breakdown results in abnormal activation of glomerular cells, formation of local scar tissue, glomerular capillaries occlusion, nephron perfusion changes, and glomerulosclerosis in final, and meanwhile, the persistent serum protein leakage damages the renal tubular epithelium, eventually leading to tubular dysfunction and tubulointerstitial fibrosis (16, 17). Until now, a considerable number of patients with proteinuria during treatment had been reported in numerous surufatinib clinical trials (4, 5, 18–20), but the mechanism of GFB breakdown remains to be further investigated.

​Several studies have reported nephrotoxicity associated with surufatinib following its approval for treatment. Zhu et al. (21) reported a case of nephrotic syndrome after surufatinib treatment and the renal biopsy confirmed thrombotic microangiopathy (TMA). Similarly, sunitinib, an oral kinase inhibitor that targets VEGF receptors 1–3 and platelet-derived growth factor (PDGF) receptors, had been approved for patients with advanced progressive pNETs over a decade (22, 23). Azar et al. (24) report a case of a patient with persistent hematuria after sunitinib (50 mg daily at bedtime) treatment for two weeks and the renal biopsy demonstrated acute interstitial nephritis (AIN). Bollee et al. (25) report a case of proteinuria and high blood pressure after sunitinib (37.5 mg daily on a 4/2 schedule) treatment for six months and the renal biopsy showed typical features of TMA. Ko et al. (26) report a case of proteinuria after sunitinib (25–50 mg daily on a 3/1 schedule) treatment for five months in a patient with renal transplantation and the allograft renal biopsy showed FSGS, AIN, and acute tubular injury. Takahashi et al. (27) report a case of nephrotic syndrome and renal dysfunction after sunitinib (25–50 mg daily on a 4/2 schedule) treatment for six months and the renal biopsy showed endothelial cell injury and FSGS. Zonoozi et al. (28) report a case of nephrotic syndrome after sunitinib treatment two years and the renal biopsy showed membranous nephropathy with PLA2R-positive immunofluorescence. These reports have documented the nephrotoxicity with the varying clinical manifestations of surufatinib or sunitinib, which target VEGF and related signaling pathways.

This case is unique in that the amelioration of nephrotic syndrome is associated with the discontinuation of surufatinib and the renal biopsy results. Based on known studies, pNET-induced IgA nephropathy and related manifestations are unlikely. Notably, the clinical manifestations of this case, particularly proteinuria, are so closely related to surufatinib treatment that drug-related nephrotic syndrome needs to be considered. However, the renal biopsy revealed IgA nephropathy with mesangial cell proliferation, moderate podocyte injury, mild renal tubule, and interstitial lesions, but no fibrosis or TMA. Nephrotic syndrome is an infrequent manifestation of IgA nephropathy, occurring in only 5% of cases (29, 30). We also detected KM55 antibody in renal tissue but the result was negative. Based on the temporal correlation between surufatinib administration and nephropathy onset, coupled with renal biopsy findings demonstrating atypical IgA nephropathy, drug-induced renal injury was prioritized in the etiological assessment after excluding tumor-associated nephropathy features.​Therefore, we consider this case to be secondary asymptomatic IgA nephropathy and that treatment with surufatinib advances kidney disease and damages the podocytes. Given the histopathological diagnosis of atypical IgA nephropathy with type 2 diabetes mellitus, the therapeutic strategy encompassed surufatinib discontinuation coupled with renin-angiotensin system inhibitors and sodium-glucose cotransporter 2 inhibitors as first-line intervention. For patients exhibiting suboptimal response, corticosteroids (e.g., prednisone 0.5 mg/kg/day) were recommended, emerging adjunctive therapies including mineralocorticoid receptor antagonists (finerenone), endothelin receptor antagonists (sparsentan), and targeted-release budesonide (Nefecon^®^) could be considered as individualized options (30). Inspired by this, further studies are required to confirm NET-related or surufatinib-related IgA nephropathy.

In conclusion, surufatinib represents an efficacious addition in patients with well-differentiated NETs, regardless of previous antitumor therapies or tumor origin. However, safety should be considered during treatment, such as in the kidney, and the mechanism of surufatinib-induced proteinuria should be investigated further.

## Data Availability

The original contributions presented in the study are included in the article/**Supplementary Material**. Further inquiries can be directed to the corresponding author.

## Supplementary material

Supplementary Figure 1 Surufatinib treatment and clinical course. ACR: Urinary microalbumin/creatinine ratios, UMa: Urinary microalbumin, ARB: Angiotensin receptor blocker.
