# Supplementary figures and images for: Surufatinib related nephrotic syndrome in a pancreatic neuroendocrine tumor: a case report and review of literature
Source: Front Oncol. 2025 May 21;15:1546217. doi: 10.3389/fonc.2025.1546217 (PMC12133497; doi:10.3389/fonc.2025.1546217)

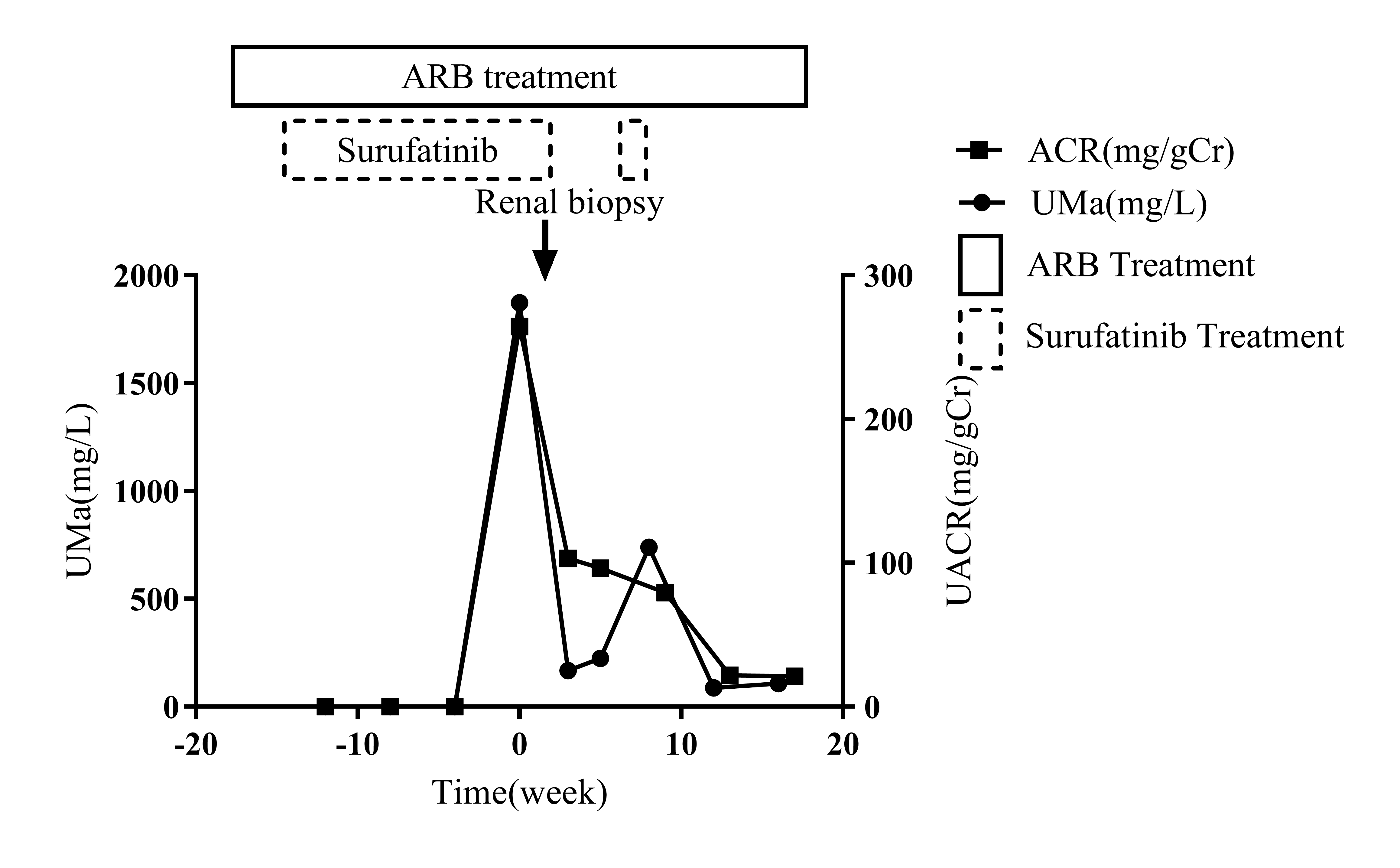

Supplement: Supplementary Figure 1 — Surufatinib treatment and clinical course. ACR: Urinary microalbumin/creatinine ratios, UMa: Urinary microalbumin, ARB: Angiotensin receptor blocker. [file Image1.tif]
